# Electrical Vestibular Stimulation after Vestibular Deafferentation and in Vestibular Schwannoma

**DOI:** 10.1371/journal.pone.0082078

**Published:** 2013-12-12

**Authors:** Swee Tin Aw, Michael John Todd, Nadine Lehnen, Grace Elizabeth Aw, Konrad Peter Weber, Thomas Eggert, Gabor Michael Halmagyi

**Affiliations:** 1 Central Clinical School, University of Sydney, Sydney, Australia; 2 Institute of Clinical Neuroscience, Royal Prince Alfred Hospital, Sydney, Australia; 3 Neurology, Ludwig-Maximilians University, German Centre for Vertigo and Balance Disorders, Munich, Germany; University of Virginia Health Science Center, United States of America

## Abstract

**Background:**

Vestibular reflexes, evoked by human electrical (*galvanic*) vestibular stimulation (EVS), are utilized to assess vestibular function and investigate its pathways. Our study aimed to investigate the electrically-evoked vestibulo-ocular reflex (eVOR) output after bilateral and unilateral vestibular deafferentations to determine the characteristics for interpreting unilateral lesions such as vestibular schwannomas.

**Methods:**

EVOR was recorded with dual-search coils as binocular three-dimensional eye movements evoked by bipolar 100 ms-step at EVS intensities of [0.9, 2.5, 5.0, 7.5, 10.0]mA and unipolar 100 ms-step at 5 mA EVS intensity. Five bilateral vestibular deafferented (BVD), 12 unilateral vestibular deafferented (UVD), four unilateral vestibular schwannoma (UVS) patients and 17 healthy subjects were tested with bipolar EVS, and five UVDs with unipolar EVS.

**Results:**

After BVD, bipolar EVS elicited no eVOR. After UVD, bipolar EVS of one functioning ear elicited bidirectional, excitatory eVOR to cathodal EVS with 9 ms latency and inhibitory eVOR to anodal EVS, opposite in direction, at half the amplitude with 12 ms latency, exhibiting an excitatory-inhibitory asymmetry. The eVOR patterns from UVS were consistent with responses from UVD confirming the vestibular loss on the lesion side. Unexpectedly, unipolar EVS of the UVD ear, instead of absent response, evoked one-third the bipolar eVOR while unipolar EVS of the functioning ear evoked half the bipolar response.

**Conclusions:**

The bidirectional eVOR evoked by bipolar EVS from UVD with an excitatory-inhibitory asymmetry and the 3 ms latency difference between normal and lesion side may be useful for detecting vestibular lesions such as UVS. We suggest that current spread could account for the small eVOR to 5 mA unipolar EVS of the UVD ear.

## Introduction

Human electrical (galvanic) vestibular stimulation has been used for over a century to probe the human vestibular system [1], [2]. The prevailing hypothesis is that electrical or galvanic vestibular stimulation evokes a vestibular response, where cathodal currents increase, and anodal currents suppress the vestibular afferent discharges posited at the spike initiation zone of the vestibular afferents [3]. Measurements of the vestibulo-ocular reflexes in response to human EVS have been extensively studied for clinical and research purposes [4]–[11]. However electrical stimulation of the vestibular system has not been widely used as a clinical test for unilateral vestibular dysfunction because the evoked response cannot be interpreted with certainty due the issues highlighted below.

One of the most intriguing findings in numerous studies is the presence of vestibular responses to bipolar (binaural) cathodal excitation of the lesion side [4], [5], [10]. These responses have been attributed either to stimulation of the vestibular afferent after an intra-labyrinthine lesion or residual functions from incomplete lesions [5], [9], [10]. However, recent studies showed that intra-labyrinthine lesion due to hair cell death from systemic gentamicin vestibulotoxicity severely impaired the eVOR [6] or from intra-tympanic gentamicin injection abolished the galvanic vestibular evoked myogenic potentials [11]. The evidence suggests that it is difficult to stimulate the vestibular afferents after intra-labyrinthine lesions. We wondered if this often measured “residual” vestibular response evoked by bipolar electrical stimulation of a unilateral lesion could instead be due to anodal inhibition of the intact labyrinth by EVS suppression of the vestibular afferent discharges as proposed by the above hypothesis on the mechanism of EVS.

Unipolar EVS has been universally used to constrain the electrical stimulation of the vestibular system to one ear [4], [5], [9]. However studies have also reported vestibular responses to unipolar electrical stimulation of the lesion ear [4], [9]. It was also found that the summation of unipolar responses from both sides in normal subjects resulted in responses greater than their bipolar vestibular responses [12]. However the possibility and extent of current spread across the cranium to the untargeted ear has not been examined. Certainly with an implantable vestibular prosthesis, great precautions have to be implemented to prevent current spread when the implant electrically stimulates the vestibular nerve [13], [14]. If current spread to the untargeted intact labyrinth does occur during human unipolar EVS, then the electrically-evoked vestibular response will include components from both ears and thus may explain the residual vestibular response evoked by unipolar stimulation of the lesion ear.

Galvanic vestibular stimulation used in most previous studies utilized longer duration stimulus of seconds to minutes to induce nystagmus and then examines the slow-phase velocity of the vestibulo-ocular reflex [4], [5], [9], [10] and/or its tonic ocular torsion [15]. Interpretations of the results were difficult because of greater intersubject variability due to the variable nature of nystagmus [16]. In addition, some studies only examine the smaller horizontal slow phase velocity [4], [5], [10] which is about quarter of the torsional slow-phase velocity [6]–[8] and constitutes only part of the total output from EVS.

In order to improve the interpretation of the vestibular response evoked by human electrical or galvanic vestibular stimulation for clinical and research purposes, it is crucial to determine the total vestibulo-ocular reflex output characteristics by studying the eVOR in three dimensions from a validated human unilateral vestibular system. Recently, we developed a novel subset of the galvanic vestibular stimulation which we term as EVS by using a transient stimulus of 100 ms (0.1 seconds) and measuring the brief eVOR response for 150 ms. The resulting eVOR was more reliable and reproducible [6]–[8] because it was not influenced by responses from other ocular motor systems such as saccade and smooth pursuit or by adaptive changes which had latencies longer than 150 ms [17]. We also examined the total eVOR output from EVS by measuring the eVOR with high temporal and spatial, binocular, three-dimensional eye movements recordings [6]–[8]. Analyses of the onset latencies, tonic and phasic eVORs from the largest torsional component further optimized our results.

The aims of our study were to use a UVD model to investigate the human total eVOR output characteristics from bipolar and unipolar EVS of a unilateral vestibular system. Firstly, we determined the eVOR after BVD to form the basis of our negative control subjects. Secondly, we examined the eVOR characteristics to human bipolar EVS validated to be from stimulating one functioning ear after UVD. Thirdly, we ascertained if unsuspected current spread to the untargeted intact ear during unipolar EVS of the lesion ear was substantial enough to invalidate it as a unilateral test and also examined the interpretations of the unipolar EVS. Finally we investigated the eVOR in UVS to determine if human EVS was useful for detecting unilateral vestibular dysfunction. The total vestibulo-ocular reflex output characteristics from this study will improve interpretation of galvanic or electrical stimulation of the vestibular system rendering it more useful for clinical and research studies.

## Materials and Methods

### Ethics statement

The protocols were approved by Ethics Committee for University of Sydney, SLHD for Royal Prince Alfred Hospital and Ludwig Maximilian University (HREC Approval Number: 10480, Protocol No X07-0257) in accordance with the Helsinki II Declaration. All participants provided their written informed consents and the Ethics Committees approved this consent procedure.

### Subjects

Subjects were recruited and tested in Royal Prince Alfred Hospital Australia and Ludwig-Maximilians University, Germany. The following subjects were tested with binocular dual-search coils in Royal Prince Alfred Hospital. Twelve UVD patients after unilateral neurectomy of the vestibulo-cochlear nerve for UVS (side of surgery: 10 left and 2 right, 6 males and 6 females, age range = 38–87 years, *M* = 54.8 years, *SD* = 14.4) were grouped as right UVDs with left functioning ears. The surgical approach used to remove the unilateral vestibular schwannoma in the UVDs was either the retrosigmoid (suboccipital) craniotomy or middle fossa craniotomy. All patients had prior neurological consultations before surgeries to exclude possible or significant brainstem compression. The UVD patients were tested more than a year post-surgery (*M* = 8.1 years, *SD* = 5.1), and they were clinically compensated from their acute peripheral vestibulopathy. After UVD, all patients had complete hearing loss and half had facial nerve palsy on the operated side. The UVD patients did not response to the caloric test with ice irrigation on the operated side. Four UVS patients confirmed by magnetic resonance imaging (MRI) (side of lesion: 4 right, 2 males and 2 females, age range = 50–74 years, *M* = 66.0 years, *SD* = 10.9) were also tested. All UVS patients had caloric canal paresis (*M* = 100%) and ipsilesional pure-tone sensorineural hearing loss with a characteristic down-sloping high frequency hearing loss. Results were compared to 17 normal subjects (3 females and 14 males, age range = 25–70 years, *M* = 39.6 years, *SD* = 16.9) previously published [6]. Five patients after BVDs for bilateral vestibular schwannomas due to neurofibromatosis Type II (4 females and 1 male, age range = 39–60 years, *M* = 46.4 years, *SD* = 8.2), who did not have any functioning ear were tested with monocular dual-search coils in Ludwig-Maximilians University [18]. All BVDs also have bilateral sensorineural deafness and bilateral facial nerve palsies.

### Recording systems

At Royal Prince Alfred Hospital, binocular three-dimensional (3D) torsional (*x*), vertical (*y*) and horizontal (*z*) eye positions evoked by EVS were recorded using pre-calibrated dual-search coils (Skalar, The Netherlands) in supine subjects viewing a fixed target at 600 mm. The subject's head was centered in the transmitter field coils (660 mm3, 66 kHz and 100 kHz, CNC Engineering, USA) during the recording. Search-coil and current-switch signals were sampled at 5 kHz with 24-bit resolution (National Instruments, USA) with Labview (National Instruments, USA) [6]–[8]. Resolution of the recording system was 0.1 arcminute for horizontal and vertical components and 0.3 arcminute for torsional component. Maximum errors and cross-coupling were <2%. At Ludwig-Maximilians University, five BVD subjects were similarly tested and monocular 3D eye positions evoked by EVS were recorded with dual-search coils at 4 kHz with 16-bit resolution [18].

### Electrical vestibular stimulation (EVS)

Bipolar EVS comprised 100 ms (0.1sec) step of direct current at intensities of [0.9, 2.5, 5.0, 7.5, 10.0]mA delivered from a DS5 - Isolated Bipolar Constant Current Stimulator (Digitimer, UK) via 9 cm^2^ surface transmastoid electrodes in left-cathode/right-anode (lc/ra) or right-cathode/left-anode (rc/la) EVS configuration [6]–[8]. Each subject was tested with 60 repetitions at each current intensity of the bipolar EVS delivered at 1 Hz. Correlation of the eVOR to 5 mA bipolar EVS duration was tested in three normal subjects at 10–100 ms in 10 ms duration increments. The eVOR response to stimulation frequency was tested with 1 ms step of 5.0 mA bipolar EVS delivered at [1, 50, 100, 200]Hz for 100 ms in three UVD subjects.

Unipolar EVS comprising 100 ms (0.1 sec) step of 5 mA current from the DS5 delivered with electrodes placed on one mastoid and the 7^th^ cervical vertebra (C_7_) in: left-cathode/C_7_-anode (lc/C_7_a), C_7_-cathode/left-anode (C_7_c/la), right-cathode/C_7_-anode (rc/C_7_a) and C_7_-cathode/right-anode (C_7_c/ra) EVS configurations were tested in five UVDs to examine the possibility of current spread to the untargeted ear opposite to the unipolar EVS.

### Data analysis

Using automated Labview software, the data after removal of any trials with blinks were averaged, filtered and computed in 3D in rotation vectors & Euler angles as eye position, velocity and acceleration in space-fixed coordinates. Leftward, downward and clockwise directions from subject's view were positive for horizontal (*z*), vertical (*y*) and torsional (*x*) eye rotations. The eVOR latency was defined as the interval between EVS onset and when torsional velocity first exceeded 1 SD of its baseline noise. Tonic eVOR was the mean torsional velocity during a 30 ms period, 70 ms after EVS onset. Phasic eVOR was the first mean peak torsional acceleration at initiation and cessation of the EVS [6]. A generalized logistic function *V* = *a*+{(*k−a*)/[1+*qe^−b^* ^(*S*−*m*)^]^1/*v*^} was used to fit an asymmetrical sigmoid curve to the input-output relationship of the EVS to the tonic or phasic eVOR in UVDs where *S* is the EVS input and *V* is the tonic or phasic eVOR output [19].

### Statistical analysis

Statistical analysis of the data was performed with S+ (SolutionMetrics, Australia) and group means (*M*), standard error of means (*SEM*) and standard deviation (*SD*) of the eVOR for each current intensity of [0.9, 2.5, 5.0, 7.5, 10.0]mA were determined for BVD, UVD and normal subjects while group means were determined for UVS patients. Student's *t*-test for differences between two means of independent or dependent observations was used to test for differences between results. A significance (alpha) level of *p* = 0.05 was used in the statistical analysis. The statistics were reported in APA format.

## Results

### Normal eVOR

**Figure 1:**
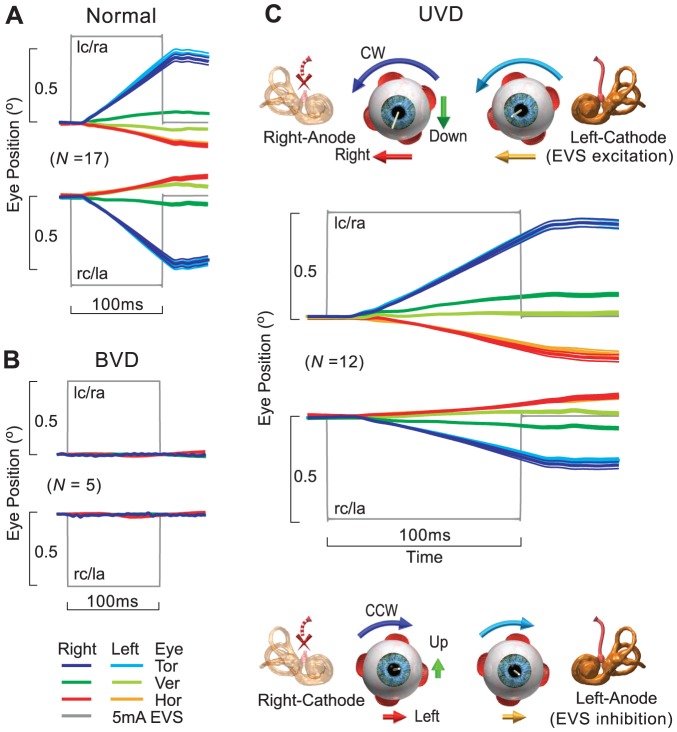
EVORs to human bipolar EVS in healthy subjects, bilateral and unilateral vestibular deafferented patients. (A) Normal eVOR from healthy subjects (*N* = 17) (group means ± SEM) comprised conjugate torsional and horizontal eye rotations, binocularly equal in amplitude, rotated away from cathode towards anode, and vertical divergence with the intorting eye upwards (cathode side) and extorting eye downwards (anode side). (B) EVOR was absent from BVD patients (*N* = 5) with torsional, vertical and horizontal eVOR positions ≤0.01°. (C) Bidirectional eVOR from grouped as right UVD patients with left functioning ear (*N* = 12). The excitatory eVOR to left-cathode/right-anode (lc/ra) cathodal EVS comprised conjugate torsional and horizontal eye rotations away from cathode and a vertical divergence with the eye on the anodal side moving downwards. The inhibitory eVOR to right-cathode/left-anode (rc/la) anodal EVS was in the opposite direction and at about half the amplitude of the excitatory eVOR with the eye on the anodal side moving upwards. (The schemes illustrate EVS polarities and eye rotation directions).

Normal eVOR comprised conjugate torsional (*x*) and horizontal (*z*) eye rotations, binocularly approximately equal in amplitude, and rotated away from the cathode towards the anode, and vertical (*y*) divergence with the intorting eye upwards on the cathodal side and the extorting eye downwards on the anodal side from lc/ra and rc/la EVS configurations (Figure 1A). Torsional eVOR was about four times larger than vertical or horizontal component [6]. Normal eVOR positions during lc/ra 5.0 mA EVS for left eye were (*x*/*y*/*z*: *M* = 0.39/−0.05/−0.10, *SEM* = 0.07/0.01/0.03)° and right eye were (*x*/*y*/*z*: *M* = 0.41/0.07/−0.10, *SEM* = 0.07/0.01/0.02)°; and during rc/la 5.0 mA EVS for left eye were (*x*/*y*/*z*: *M* = −0.42/0.08/0.09, *SEM* = 0.08/0.01/0.02)° and right eye were (*x*/*y*/*z*: *M* = −0.40/−0.03/0.07, *SEM* = 0.08/0.01/0.02)°. Normal tonic eVOR was (*M* = 5.2°/s, 95% CI[3.4, 7.0]) during lc/ra 5 mA EVS, while normal phasic eVOR initiation was (*M* = 1044°/s^2^, 95% CI[711, 1373]) and cessation was (*M* = −1090°/s^2^, 95% CI[−544, −1636]) derived from the torsional component.

### Absent eVOR after BVD

We tested five BVD patients as our negative controls, to establish whether there was any eVOR in patients without any functioning ear after complete bilateral vestibulo-cochlear nerve sections for surgical removal of vestibular schwannomas and whether EVS excited any residual vestibular nerve or the central vestibular pathways. We showed that the eVOR was completely abolished from all BVDs with the mean torsional (*x*), vertical (*y*) and horizontal (*z*) eVOR positions at ≤0.01° (Figure 1B). The eVOR positions during lc/ra 5 mA EVS for left eye were (*x*/*y*/*z*: *M* = 0.00/−0.01/0.00, *SEM* = 0.01/0.01/0.00)° and during rc/la EVS for left eye were (*x*/*y*/*z*: *M* = −0.01/0.00/0.01, *SEM* = 0.01/0.01/0.00)°. Here we verified that there was a complete loss of eVOR after BVD, when there was no functioning ear. We also confirmed that human EVS did not stimulate any residual vestibular nerve or the central vestibular pathways.

### Bidirectional eVOR after UVD

After establishing that BVD caused a complete bilateral vestibular loss with absent eVOR, likewise UVD would cause a complete unilateral vestibular loss. Therefore, we validated that any eVOR measured after UVD was from EVS of one functioning ear. Figure 1C shows the eVOR from 5 mA EVS of 12 (grouped as) right UVDs with left functioning ears. Cathodal lc/ra EVS of the left ear elicited excitatory eVOR positions comprising binocular and conjugate torsional (*x*) and horizontal (*z*) eye rotations away from left cathode, and a vertical (*y*) divergence with the eye on the anodal side moving downwards. The eVOR positions from the left eye were (*x*/*y*/*z*: *M* = 0.40/0.00/−0.15, *SEM* = 0.07/0.03/0.05)° and right eye were (*x*/*y*/*z*: *M* = 0. 39/0.06/−0.15, *SEM* = 0.09/0.03/0.06)°. Anodal rc/la EVS of the left ear elicited inhibitory eVOR positions of about half the excitatory amplitude, comprising binocular and conjugate torsional (*x*) and horizontal (*z*) eye rotations towards left anode and a vertical (*y*) divergence with only the eye on the right cathodal side moving upwards. The eVOR positions from the left eye were (*x*/*y*/*z*: *M* = −0.19/−0.01/0.07, *SEM* = 0.03/0.02/0.02)° and right eye were (*x*/*y*/*z*: *M* = −0.22/−0.06/0.06, *SEM* = 0.07/0.02/0.02)°. We showed that eVORs from UVDs with one functioning left ear was a bidirectional, excitatory eVOR to cathodal EVS and inhibitory eVOR to anodal EVS. The inhibitory eVOR was in the opposite direction to and at about half the amplitude of the excitatory eVOR, exhibiting excitatory-inhibitory asymmetry. In contrast to normal eVOR, the eye ipsilateral to the functioning ear did not generate any vertical (*y*) component to EVS.

### Effects of EVS intensity, duration and frequency on the eVOR after UVD

**Figure 2:**
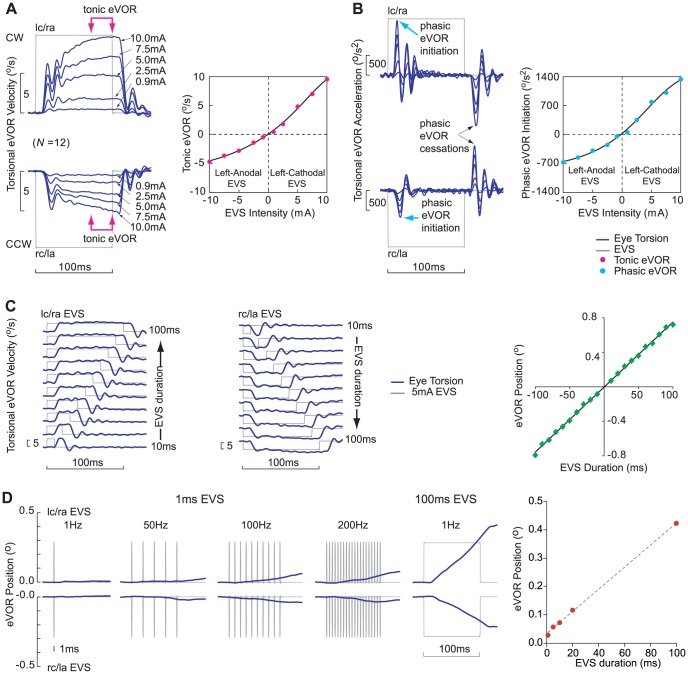
Spatio-temporal characteristics of the eVOR to human bipolar EVS. (A, B) Mean eVOR velocity and acceleration time-series to EVS intensities of [0.9, 2.5, 5.0, 7.5, 10.0]mA from grouped as right UVDs with functioning left ears (N = 12). Tonic and phasic eVORs graded to all current intensities for lc/ra cathodal EVS excitation or rc/la anodal EVS inhibition exhibiting excitatory-inhibitory asymmetries. (C) Relationship of eVOR with EVS duration and frequency. Normal eVOR was linearly correlated with EVS duration when EVS duration was incremented from 10–100 ms in 10 ms-step. (D) Comparison of excitatory and inhibitory eVOR positions to a 1 ms EVS step at [1, 50, 100, 200]Hz and to a 100 ms EVS step at 1 Hz. We found that the eVOR correlated well to EVS duration but not to frequency of stimulation.

We examined the tonic and phasic eVORs in response to excitatory and inhibitory EVS intensities in grouped as right UVDs with functioning left ears. Tonic and phasic eVOR had spatio-temporal properties [6] as illustrated by the spatial torsional (*x*) component. After EVS onset and offset, there were phasic eVORs i.e. the phasic initiation and cessation acceleration pulses, whereas during the 100 ms of constant current stimulation there was a tonic eVOR i.e. the approximately constant 30 ms of eye velocity at 70 ms after onset [6]. Both tonic and phasic eVORs graded to cathodal excitation and anodal inhibition of the functioning left ear to all EVS intensities. The mean inhibitory tonic and phasic eVORs in UVDs was about half the excitatory eVORs and exhibited the excitatory-inhibitory asymmetries (Figure 2). A generalized logistic function [19] was used to fit an asymmetrical sigmoid curve to the input-output relationships in UVDs between the cathodal and anodal EVS inputs with their tonic or phasic eVOR outputs in Figures 2A and 2B.

Comparison of the tonic eVOR after UVD to normal showed that tonic eVOR for the left ear from cathodal lc/ra EVS intensities of [0.9, 2.5, 5.0, 7.5, 10.0]mA were (*M* = 0.5, 1.8, 4.8, 7.0, 9.5°/s, *SD* = 0.4, 1.1, 2.8, 3.8, 4.8) and were not different from normal eVOR, *t*(27) = 2.17, 1.51, 0.43, 0.02, 0.27, *p* = 0.06, 0.16, 0.68, 0.98, 0.79. Tonic eVOR from anodal rc/la EVS of left ear were (*M* = −0.3, −1.4, −2.8, −3.7, −5.0°/s, *SD* = 0.6, 1.1, 2.3, 3.0, 3.4) and were lower than normal eVOR, *t*(27) = 3.00, 2.41, 2.92, 3.71, 4.19, *p* = 0.01, 0.03, 0.01, 0.00, 0.00. Comparison of tonic eVOR between intact and UVD side showed that tonic eVOR of the left ear from cathodal EVS at intensities of [5.0, 7.5, 10.0]mA were (*M* = 4.8, 7.0, 9.5°/s, *SD* = 2.8, 3.8, 4.8) and higher than for anodal EVS (*M* = −2.8, −3.7, −5.0°/s, *SD* = 2.3, 3.0, 3.4), *t*(11) = 5.25, 5.46, 5.12, *p* = 0.00, 0.00, 0.00. The excitatory-inhibitory asymmetry ratio of the tonic eVOR to EVS of [0.9, 2.5, 5.0, 7.5, 10.0]mA were [1.0, 1.2, 1.7, 1.9, 2.0] respectively. Spatially the initial excitatory eVOR velocity profile had double peaks compared to the single peak in the inhibitory eVOR and they were both different from normal eVOR velocity.

Comparison of the phasic eVOR after UVD to normal showed that phasic eVOR initiation for the left ear from cathodal EVS intensities of [0.9, 2.5, 5.0, 7.5, 10.0]mA were (*M* = 101, 349, 789, 994, 1338°/s^2^, *SD* = 100, 253, 349, 390, 526) and were not different from normal, *t*(27) = 2.08, 2.13, 1.79, 0.58, 0.35, *p* = 0.06, 0.06, 0.10, 0.57, 0.73. Phasic eVOR initiation from anodal EVS of the left ear were (*M* = −40, −232, −353, −540, −652°/s^2^, *SD* = 114, 147, 192, 227, 325) and were lower than normal, *t*(27) = 3.24, 3.60, 7.40, 4.76, 5.13, *p* = 0.01, 0.00, 0.00, 0.00, 0.00. Comparison of phasic eVOR between intact and UVD side for EVS intensities of [5.0, 7.5, 10.0]mA showed that phasic eVOR from cathodal EVS of left ear were (*M* = 789, 994, 1338°/s^2^, *SD* = 349, 390, 526) were higher than for anodal EVS (*M* = −353, −540, −652°/s^2^, *SD* = 192, 227, 325), *t*(11) = 7.32, 6.80, 3.94, *p* = 0.00, 0.00, 0.00 (Figure 2B).

We quantified the effect on eVOR position from 5 mA EVS of step durations from 10–100 ms in 10 ms increment in 3 normal subjects (Figure 2C). We showed a linear input-output relationship with a correlation of (*R^2^* = 0.99) between the EVS duration input to the eVOR position output. We examined the effect of increasing stimulation frequency in UVDs with a functioning left ear with a 5.0 mA, 1 ms EVS step (Figure 2D). When this 1 ms EVS was delivered at 1 Hz, 50 Hz, 100 Hz and 200 Hz for 100 ms duration the eVOR positions increased as the stimulation frequency increased. Surprisingly, the eVOR position achieved at 200 Hz was still smaller than the eVOR from a 100 ms EVS at 1 Hz. Analysis showed that eVOR position was proportional to EVS duration but not to stimulation frequency.

### Latency of eVOR

**Figure 3:**
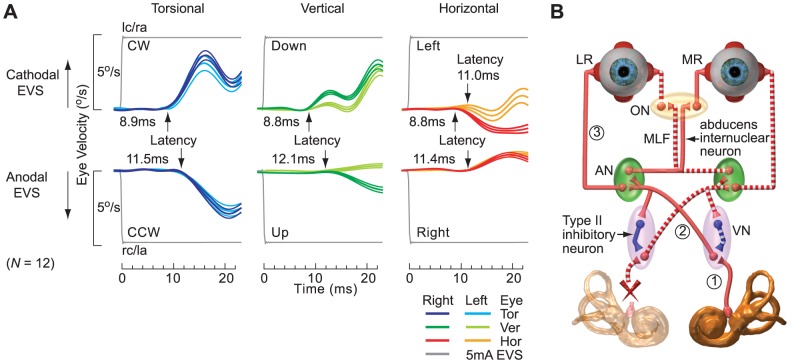
Latency of the eVOR after UVD. (A) Excitatory and inhibitory eVOR latencies of binocular torsional, vertical and horizontal eVOR to 5.0 mA lc/ra cathodal EVS and rc/la anodal EVS from grouped as right UVDs with functioning left ears (*N* = 12, group means ± SEM) showing their mean latencies. (B) Schematic depicting the excitatory (*solid red line*) and inhibitory (*dashed red line*) horizontal semicircular canal pathways showing the 3-neuron reflex arc comprising **1**: vestibular nerve; **2**: vestibulo-ocular secondary neuron; **3**: abducens motorneuron. LR: lateral rectus muscle; MR: medial rectus muscle; ON: oculomotor nucleus; AN: abducens nucleus; VN: vestibular nucleus; MLF: medial longitudinal fasciculus. Equivalent 3-neuron reflex arcs also exist for the vertical semicircular canal pathways.

After UVD, the excitatory eVOR latency to 5 mA EVS from the left functioning ear (grouped as right UVDs) were (*binocular x*/*y*: *M* = 8.9/8.8 ms, *SD* = 0.2/0.2; *ipsilateral/contralateral z*: *M* = 11.0/8.8 ms, *SD* = 0.2/0.3) (Figure 3A). This excitatory eVOR latency from left functioning ear after right UVD was the same as normal latency [6], [8] determined using the torsional component of (*binocular x*: *M* = 8.9 ms, *SD* = 0.3), *t*(11) = 0.41, *p* = 0.69. The mean inhibitory eVOR latency of (*binocular x*/*y*/*z*: *M* = 11.6/12.1/11.4 ms, *SD* = 0.2/0.2/0.2) were longer than excitatory eVOR latencies *t*(11) = 2.20, *p* = 0.00. Figure 3B is the schematic depicting the excitatory (*solid red line*) and inhibitory (*dashed red line*) horizontal semicircular canal pathways showing the 3-neuron reflex arc.

### Unipolar EVS

**Figure 4:**
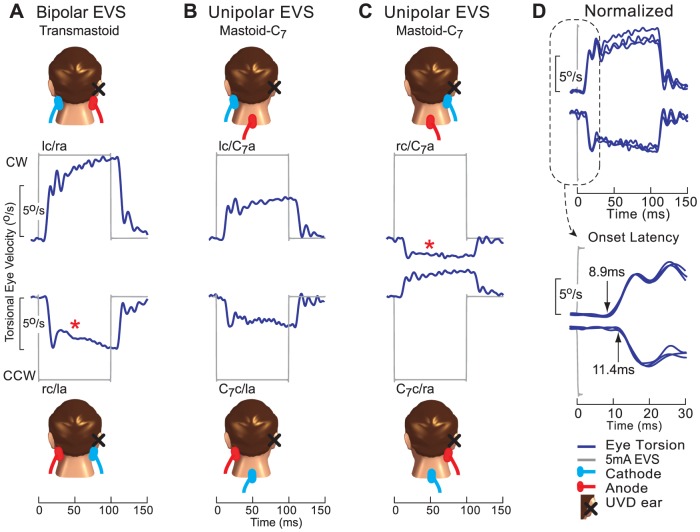
Effect of current spread in human unipolar EVS of UVD patients. The eVOR to unipolar 5.0(A) The eVORs from left-cathode/right-anode (lc/ra) and to right-cathode/left-anode (rc/la) bipolar EVS were largest. (B) When left-cathode/C_7_-anode (lc/C_7_a) and C_7_-cathode/left-anode (C_7_c/la) unipolar EVS stimulated the functioning left ear, the eVOR was about half the bipolar eVOR. (C) However when right-cathode/C_7_-anode (rc/C_7_a) and C_7_-cathode/right-anode (C_7_c/ra) unipolar EVS stimulated the right UVD ear, instead of the expected absent response, it was one-third the bipolar eVOR. The polarity of the eVOR was consistent with the C_7_- electrode polarity suggesting that current may have spread from the C_7_-electrode closer to functioning left ear. (D) When the eVOR from all three configurations were grouped according to direction of their responses (i.e. CW or CCW) and then normalized, they share similar spatio-temporal characteristics with the mean excitatory eVOR latency of 8.9 ms and inhibitory latency of 11.4 ms suggesting that the eVORs were generated from the functioning left ear. (Schemes show electrode locations on the patient).

We investigated whether unipolar EVS is a unilateral stimulation by testing UVDs with one validated functioning ear to examine the effects of possible current spread during EVS. We used the EVS intensity of 5 mA and the most commonly used unipolar galvanic stimulation configuration between the mastoid and C_7_ vertebra. Five UVDs grouped as right UVDs with left functioning ear were tested with 5 mA unipolar EVS using electrodes placed over one mastoid bone referenced to another on C_7_ vertebra. Figure 4 compares the tonic eVOR from bipolar EVS with unipolar EVS of the left functioning ear and right UVD ear. Tonic eVORs from bipolar EVS were (lc/ra: *M* = 7.97°/s, *SD* = 0.04; rc/la: *M* = −4.94°/s, *SD* = 0.02) (Figure 4A). Tonic eVORs from unipolar EVS of left ear were (lc/C_7_a: *M* = 3.80°/s, *SD* = 0.04; C_7_c/la: *M* = −2.70°/s, *SD* = 0.02) (Figure 4B) and right UVD ear were (ra/C_7_c: *M* = 2.39°/s, *SD* = 0.02; rc/C_7_a: *M* = −1.80°/s, *SD* = 0.02) (Figure 4C). We found that unipolar EVS of left functioning ear was half the bipolar eVOR. Surprisingly we found that unipolar EVS of the right UVD ear produced one-third the bipolar eVOR, instead of the expected absent response. The direction of this eVOR could be predicted by the C_7_ electrode polarity which was the closer electrode to the left functioning ear.

When the eVOR from all three configurations were grouped according to direction of their responses (*i.e. CW or CCW*) and then normalized, their spatio-temporal correspondence suggested that each response were generated from the same stimulus polarity of the same ear (Figure 4D). The excitatory eVOR latency was (*M* = 8.9 ms, *SD* = 0.2) and the inhibitory eVOR latency was (*M* = 11.4 ms, *SD* = 0.2). Therefore the different electrode positions modulated the current intensity stimulating the functioning left ear.

### eVOR in UVS

**Figure 5:**
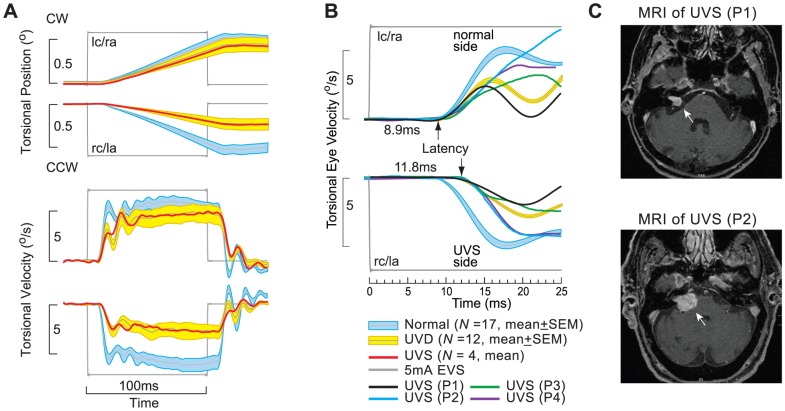
Comparison of the mean eVOR in UVS to normal and UVD subjects. (A) The mean eVOR in UVS (*N* = 4) was similar to UVD grouped as right lesions, but different from normal. Right UVS showed bidirectional, excitatory eVOR to 5 mA cathodal EVS and inhibitory eVOR to anodal EVS of the left ear at half the amplitude and opposite in direction, with excitatory-inhibitory asymmetrical response pattern similar to the right UVD. Mean latencies from 4 individual UVS patients (P1, P2, P3, P4) were compared to normal and right UVD subjects. (C) MRI from UVS patients (P1, P2)

Vestibular schwannoma compresses the vestibulo-cochlear nerve causing diminution and loss of vestibular function. The eVOR from the four UVS patients were grouped as right UVS and averaged. The mean torsional eVOR positions and velocities to 5 mA EVS from right UVS were compared to normal and grouped right UVD (Figure 5A). Right UVS showed bidirectional, excitatory eVOR to 5 mA cathodal EVS (*M* = 4.35°/s) and inhibitory eVOR to anodal EVS (*M* = −2.68°/s) of the left ear at half the amplitude and opposite in direction, with excitatory-inhibitory asymmetrical response pattern similar to the right UVD. Figure 5B illustrates the mean eVOR onset latency to 5 mA EVS from four UVS patients (displayed as right UVS) by comparing their mean torsional eVOR velocity at onset to normal and UVD eVOR latencies. The mean eVOR onset latency in UVS for bipolar cathodal EVS of normal left side was (*x: M* = 8.9 ms, *SD* = 0.2) and for right UVS side was (*x: M* = 11.8 ms, *SD* = 0.4). Figure 5C shows two examples of vestibular schwannoma lesions on MRI.

## Discussion

Our study of the total vestibulo-ocular reflex output characteristics to human bipolar and unipolar EVS using our validated UVD model showed two novel results. Firstly, EVS of one intact ear after UVD generates *bidirectional* eVORs. Cathodal EVS evokes excitatory eVOR and anodal EVS evokes inhibitory eVOR, exhibiting an excitatory-inhibitory asymmetry and a 3 ms latency difference between excitatory and inhibitory response. Secondly, *current spread* during unipolar EVS activates both ears, demonstrating that it is not an entirely unilateral stimulation.

### Absent eVOR after BVD

In order to use human EVS to detect unilateral vestibular pathology, it is crucial to determine the electrically-evoked vestibular response emanating from a validated unilateral vestibular system. We established our negative controls by testing BVD patients who had undergone vestibular deafferentation for surgical removal of bilateral vestibular schwannomas, to ensure that when bilateral vestibular deafferentations of their vestibulo-cochlear nerves were complete, there was no eVOR to EVS. All BVD patients also had bilateral sensorineural hearing loss. We showed a complete bilateral eVOR loss after BVD (Figure 1B). This ascertained that no eVOR was generated from EVS of the ear after vestibular deafferentation. Therefore after UVD, the eVOR from EVS must originate only from the remaining functioning ear with an intact vestibular labyrinth and nerve. We also confirmed that human EVS did not stimulate any residual vestibular nerve or the central vestibular pathways.

### Bidirectional eVORs after UVD

After UVD, eVORs from EVS of one functioning ear were bidirectional, excitatory eVOR to cathodal EVS and inhibitory eVOR to anodal EVS at half the amplitude and opposite in direction, with an excitatory-inhibitory asymmetry. This bidirectional eVOR from bipolar EVS of a unilateral vestibular system can be explained by cathodal excitation which increases the firing rate to generate an excitatory response while anodal inhibition suppresses the firing rate to generate an inhibitory response [3] (*which is anodal EVS of the intact ear in either bipolar or unipolar configuration, see* ★ *in* *Figure 4*). The excitatory-inhibitory asymmetry can be attributed to the anodal EVS driving the vestibular afferent discharge towards inhibitory cut-off which puts the vestibular afferent response into the non-linear operating range [20]. In addition, the initial excitatory eVOR velocity profile had double peaks and was spatially different to the single peak in the inhibitory eVOR (Figure 2A) and they were both different from normal eVOR velocity (Figure 5A). The inhibitory response may be been mistaken for the residual response from the lesion ear if it was examined based only on the response direction.

### Effects of EVS intensity, duration and frequency on the eVOR after UVD

In order to determine the optimal EVS that is useful for clinical and research studies, we examined the relationships in UVDs between the EVS inputs and their eVOR outputs. Torsional component is the largest and most sensitive component being four times larger than horizontal or vertical component. Both tonic and phasic eVOR outputs showed asymmetrical sigmoid relations to EVS intensity (Figure 2). The excitatory-inhibitory asymmetry ratio of the eVOR to EVS of [0.9, 2.5, 5.0, 7.5, 10.0]mA were [1.0, 1.2, 1.7, 1.9, 2.0] respectively. These results suggest bipolar electrical or galvanic vestibular stimulation of ≥5 mA will induce an eVOR excitatory-inhibitory asymmetry to comfortably detect the lesion side. The eVOR output is linearly correlated to EVS duration rather than to EVS frequency. Therefore increasing the duration of the EVS current-step to 100 ms has increased the eVOR peak position thus improving its sensitivity, without being long enough to induce nystagmus or be influenced by saccades [17].

### eVOR latency

Measurement of eVOR onset latencies in UVD with one functioning ear elucidated the precise timings required to traverse the excitatory and inhibitory central vestibular pathways. The advantage of using EVS rather than a mechanical vestibular stimulus was that the instantaneous EVS onset circumvented latency inaccuracies due to head inertia and movement artifact. After UVD, we showed that the inhibitory eVOR latency is 3 ms longer than the excitatory latency. The excitatory eVOR latency from cathodal EVS of the functioning ear was 9 ms for binocular torsional (*x*), vertical (*y*) and contralateral horizontal (*z*) eye rotations, which were similar to normal eVOR latency [6], [8] (Figure 3). The excitatory torsional eVOR latency was also similar to mechanically-evoked vestibulo-ocular reflex latency [21]. However, the excitatory ipsilateral horizontal (*z*) latency to the functioning ear was 11 ms. The 2 ms latency increase could be explained by the extra abducens internuclear neuron and synapse which connects the contralateral abducens nucleus with the ipsilateral oculomotor nucleus through medial longitudinal fasciculus [22] (Figure 3B). The mean inhibitory eVOR latency from all the eye rotation (*horizontal, vertical and torsional*) components was 12 ms. This 3 ms latency increase could be explained by the extra medial vestibular nucleus Type II inhibitory neuron and synapse of the commissural inhibitory vestibular pathway [23] and also longer inhibitory mediation [24]. Also this slower inhibitory eVOR latency cannot be mistaken as cathodal excitation of any residual function from the right UVD ear which would produce a 9 ms latency.

### Unipolar EVS

We showed that unipolar EVS was not a truly unilateral test. During unipolar EVS, the prospect of current spread across the head to the untargeted opposite ear has historically been discounted [4], [5], [9], [10], [12]. We used a widely accepted mastoid-7^th^ cervical vertebral configuration for our unipolar EVS [4], [5], [9], [16] and also a 5 mA EVS, a current commonly used for galvanic stimulation [4], [16]. We found that the eVOR from unipolar EVS of the functioning ear was half its bipolar eVOR (Figure 4B). However, unipolar EVS of the UVD ear showed one-third the bipolar eVOR instead of the expected absent eVOR (Figure 4C). Since we validated that no eVOR was generated from the UVD ear, this response must have originated from the untargeted contralateral functioning ear. This was confirmed by the similarities in spatial response patterns and latencies when the responses from bipolar and unipolar EVS were grouped according to response directions and normalized (Figure 4D). The polarity of the C_7_-electrode which was closer to the functioning ear determined this eVOR direction. The vestibulo-ocular reflex response that we measured during unipolar EVS of the UVD ear did not arise from the inadvertent ipsilateral cervical vestibulospinal tract stimulation because the medial vestibulospinal tract innervates the neck muscles that support the head and such stimulation would evoke head and neck movements instead of the eye movements that we found.

Consequently, this current spread stimulating the untargeted functioning ear would cause the summation of unipolar responses from both sides in normal subjects to be greater than the bipolar vestibular response [12]. Notwithstanding the current spread issue, in clinical studies there would still be an asymmetrical response between the intact and lesion sides which would be useful for determining the side of the pathology (Figures 4B and 4C, top panels). However, interpretations of the vestibular responses to unipolar EVS using normal subjects in models of the vestibular pathways [12] may require reassessments with UVD subjects.

### eVOR in UVS

UVS highlighted the kind of unilateral vestibular pathology that the human EVS may be used to test. We showed that the eVOR response patterns from UVS were similar to that from UVD, conforming to the excitatory-inhibitory eVOR asymmetry and the 3 ms latency difference between intact and lesion sides suggesting that in UVS, compression of the vestibular nerve by the vestibular schwannoma caused a vestibular deficit similar to a UVD (Figure 5). The UVS patients that we presented have large vestibular schwannoma lesions consequently they were equivalently deafferented due to compression by the vestibular schwannoma. However in patients with smaller vestibular schwannoma lesions the effects may be smaller. Based on our spatio-temporal response pattern, the asymmetry between the intact and lesion ear will be present but only in a different ratio if there is residual vestibular function on the lesion side, but the latency difference of 3 ms between the intact and lesion ear may be absent or reduced.

## Conclusions

Our study elucidated the features of the vestibulo-ocular reflex evoked by electrical stimulation that will improve the usefulness of EVS as a clinical test of unilateral vestibular dysfunction. We showed that human bipolar and unipolar EVS of 5 mA or higher reliably identified a unilateral vestibular lesion, where the eVOR from the lesion ear is half of that from the intact ear. A prolonged latency of 3 ms between the lesion and intact ear suggested a complete lesion. The bipolar EVS test was twice as sensitive as the unipolar EVS test. Measurement of the torsional eye movement component had four times the sensitivity of the measuring the horizontal component.

As unipolar EVS activates both ears due to current spread, consequently unipolar EVS should not be used synonymously as a unilateral stimulation to deduce vestibulo-ocular and -spinal reflex pathways especially in normal subjects with two functioning ears. Whilst the current spread has minor implication for clinical interpretation, models of the vestibular pathways using EVS will require studies with UVD subjects.
